# Morphological and Eco-Geographic Variation in Algerian Wild Olives

**DOI:** 10.3390/plants11141803

**Published:** 2022-07-08

**Authors:** Wahiba Falek, Isabella Mascio, Susanna Gadaleta, Valentina Fanelli, Sakina Bechkri, Douadi Khelifi, Monica Marilena Miazzi, Cinzia Montemurro

**Affiliations:** 1 Ecole Nationale Supérieure de Biotechnologie, Constantine 251000, Algeria; wfalek@ymail.com (W.F.); s.bechkri@gmail.com (S.B.); dkhelifi@yahoo.fr (D.K.); 2 Department of Soil, Plant and Food Sciences, University of Bari Aldo Moro, 70125 Bari, Italy; mascioisa@gmail.com (I.M.); valentina.fanelli@uniba.it (V.F.); cinzia.montemurro@uniba.it (C.M.); 3 Spin off Sinagri s.r.l., University of Bari Aldo Moro, 70125 Bari, Italy; sanna14@hotmail.it

**Keywords:** *Olea* var. *sylvestris*, oleaster, biodiversity, bioclimatic evaluation, pluviometric variation, Algeria

## Abstract

Algerian wild olives can represent an important resource for cultivated olive breeding, since they are characterized by great morphological variability. Moreover, they grow in different bioclimatic environments, including dry and hot climates, making the collections of wild olives a good source of abiotic stress resistance traits. Our study aimed to investigate the morphological diversity of 175 wild olive trees collected in North Algeria along with a wide range of different bioclimatic habitats for studying traits of olive accessions in relation to their different ecogeographical parameters. Wild olive trees were found in five different bioclimates areas spanning from humid to Saharan areas. They showed high variation in all traits, in particular fruit and stone weight, which expressed the highest coefficient of variation, and a high positive correlation between fruit weight/width. Cluster analysis separated the samples into two groups mostly based on fruit and stone size, while no relationship was observed with the area of sampling. Only the Saharan samples showed significantly different foliar and fruit characteristics compared to samples from other bioclimatic areas.

## 1. Introduction

The olive tree (*Olea europaea* subsp. *aeuropea* L.) is an evergreen tree belonging to the *Oleaceae* family. In the Mediterranean Basin, it has high social and economic importance, being a fundamental nutritional source due to olive oil’s high levels of monosaturated fatty acids and phenolic compounds [1]. Six subspecies are known: *cuspidata*, *guanchica*, *cerasiformis*, *laperrinei*, *maroccana*, and *europaea*. In particular, wild olive trees, or oleaster (var. *sylvestris*) and cultivated olive trees (var. *europaea*) constitute the two botanical varieties of *O. europaea* [2]. Several morphological, ecological, and cytogenetic studies have shown that *O. europaea* subsp. *europaea* var. *sylvestris* is the wild relative of the cultivated olive [3].

Wild and domesticated olives grow in the same climatic area, but the wild type shows some morphological differences, such as smaller fruit size and lower oil content in the mesocarp [4]. The different pedoclimatic growing environments of wild olive include areas characterized by extreme levels of drought, high temperatures, and high salinity [5,6]. Wild olive exhibits a higher genetic diversity than the cultivated subspecies [7], holding agronomic traits, such as a shorter juvenile period and resistance to biotic and/or abiotic stress [8,9,10], which could be useful to transfer into the cultivated olive. Moreover, wild olive can be useful to improve the olive oil’s health value and taste, being richer than the cultivated olive, in antioxidants and oleic acid [11,12,13], which are components increasingly appreciated by the pharmaceutical sector and consumers.

Algeria is a large Mediterranean country that stretches over 2.4 million square kilometers, with the Atlas Mountains dividing the territory into two parts. In Northern Algeria, the coasts are generally very close to the mountains and are the most populated and cultivated areas. To the south, the main part is occupied by the Sahara desert, where other olive subspecies such as *O. europaea* subsp. *laperrinei* have been discovered and studied [14,15]. At the beginning of the 2000s, olive groves covered only 2.3% of the total cultivated area, but in recent years, the Algerian Agriculture Ministry has allocated resources for the development and support of the olive-growing sector, which has allowed increasing the area dedicated to olive growing, from 165,600 hectares in 1999 to 439,000 in 2020, and the olive production from 363,381 to 1,079,508 tonnes [16].

While only 36 cultivars are described and listed in the catalog of Algerian olive varieties [17] Algeria still preserves important reserves of wild olives, which have captured attention as a resource to diversify and enrich the country’s olive heritage [18,19]. The awareness that the availability and conservation of the wild olive germplasm suitable for breeding programs are crucially important has prompted intensifying research and conservation projects for new germplasm in all olive-producing countries [20,21,22,23]. An efficient collection of natural diversity to mine for specific traits can be obtained by focusing on natural areas characterized by extreme climates. Therefore, populations of wild olive that have remained isolated in remote areas far from the cultivated fields, could represent a good source of traits useful in olive breeding programs.

The morphological and ecogeographic characterization of the wild olive genetic resources in Algeria would provide solid information on the current state of the species and would help to define adequate conservation strategies. For this aim, other studies have already been conducted, but they involved a few samples and were collected in limited areas [18,24,25]. Today, molecular techniques are preferred for genetic diversity evaluation [26,27,28], and advances in high-throughput sequencing have made the genomes of many crops available, including wild olive [29]. Nonetheless, morphological characterization is the first step in biodiversity description and classification, and it is required to understand genotype-phenotype relationships for the development of crop breeding [30,31]. 

The aim of this study was the morphological characterization of a wide collection of wild olive samples growing in different ecoclimatic zones of Northern Algeria (i) to explore the variation of samples in relation to their ecogeographic origin, and (ii) to investigate the possibility of identifying, in specific genotypes, traits of adaptation worthy of being further studied for use in breeding programs. 

## 2. Materials and Methods

### 2.1. Field Surveys and Sampling 

**Figure 1:**
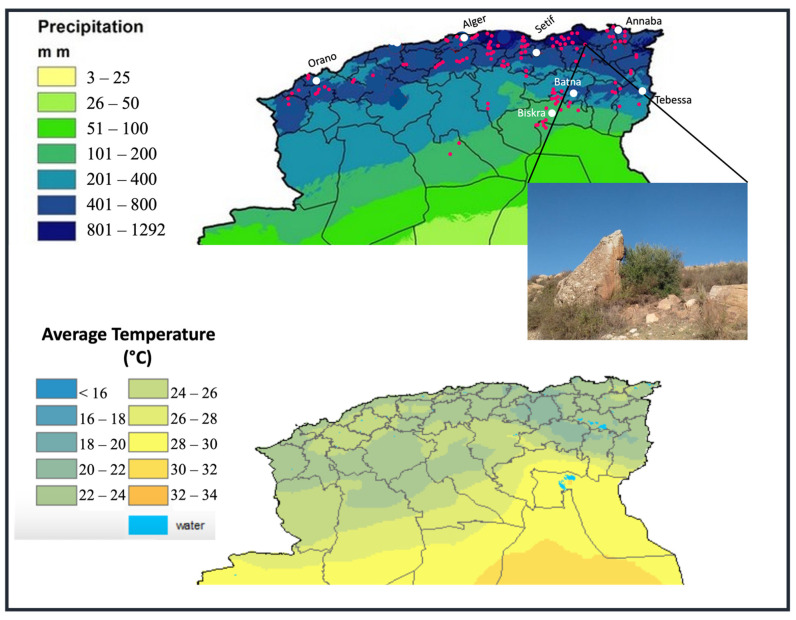
Map of Northern Algeria showing the sampling sites of wild trees. The average precipitation and temperature are also shown. The picture refers to the sample Constantine_11—Constantine, Djbel El Ouehch. Source: https://earlywarning.usgs.gov (accessed on 4 May 2022).

Prospecting and sampling were conducted during the harvest period of 2017 in Northern Algeria in a wide range of natural habitats characterized by different bioclimatic conditions (Figure 1). Thirty-four provinces located along the coast and in the inner province of Laghouat, at an altitude ranging from 18 m asl (Tipaza_1) to 1270 m asl (Batna_8), were inspected, and sampling sites were geo-referenced using a Global Positioning System (GPSGARMIN Trex^®^ model 30) (Supplementary Table S1). From each of the 175 wild olive samples, 30 leaves and 30 fruits were randomly collected in different parts of the tree and stored in refrigerated boxes until their analysis. Samples were collected from shrubs often dense, twiggy, and spiny with ovate-oblong to elliptic leaves and small drupes (<1 cm long) with fleshy but thin mesocarp and low oil content, following the methodology established by the International Union For The Protection Of New Varieties Of Plants (UPOV) for primary characterization of olive [32] (Table 1). 

### 2.2. Ecogeographic Data

For each sampling site, the altitude and the principal climate parameters, annual rainfall, average of the maximum temperature of the hottest month, and average of the minimum temperature of the coldest month, were recorded by the National Office of Meteorology of Algeria (ONM), except for thirteen locations for which the data were not available. For these locations, the data were recorded from CLIMATE-DATA.ORG (http://fr.climate-data.org/ (accessed on 1 November 2017) (Supplementary Table S1). Climate parameters were used to calculate the pluviothermic Emberger coefficient (Q2), whose values correlate with humidity levels (differential dryness) characterizing the Mediterranean climate [33]. Moreover, the pluviothermic coefficient along with the minimum temperature of the coldest month was used to obtain the Emberger’s climogram [34].

### 2.3. Morphological Quantitative and Qualitative Traits 

The morphological characterization of wild olive samples was carried out by using 32 olive descriptors indicated by UPOV and modified for wild olives, including 5 traits related to the tree, 4 descriptors for leaf, 11 for the fruit, and 12 for the stone, considering up to 4 classes in qualitative traits [6,32] (Supplementary Table S1, Supplementary Figure S1). Twenty traits were considered exclusively qualitative, 4 were considered exclusively quantitative, and 8 morphological traits were considered from both a qualitative and quantitative point of view (Table 1). 

### 2.4. Data Analysis

Quantitative traits were checked for normality of frequency distributions and variance heterogeneity and used for descriptive statistical analysis which included range, average, standard deviation, and coefficient of variation (CV%) to evaluate the amplitude of trait variability [35]. Correlation analysis among the observed variables [36] and one-way analysis of variance (ANOVA) at the significant level of *p* < 0.05 were performed using XLSTAT software (https://www.xlstat.com, accessed on 4 May 2022).

Qualitative traits data were converted into a discrete data matrix and used in the software GENALEX v.6.5 (accessed on 4 May 2022) [37]. Diversity across wild olive trees and the efficiency of descriptors in distinguishing the individuals were evaluated by calculating the Shannon information index (Hj), and the discrimination power (Dj) [38].

A principal component analysis (PCA) was also performed to identify the patterns of variation within the samples in order to determine the relative importance of the classification variables [39] and to select them for further hierarchical cluster analysis. Cluster analysis was carried out by using the software DARWIN v. 6.0.010 (http://darwin.cirad.fr, accessed on 4 May 2022) based on Ward’s minimum variance method [40], and the tree was visualized by using the software FigTree 2016-10-04-v1.4.4 (http://tree.bio.ed.ac.uk/software/figtree, accessed on 4 May 2022).

## 3. Results

### 3.1. Ecogeographic Data Analysis

**Figure 2:**
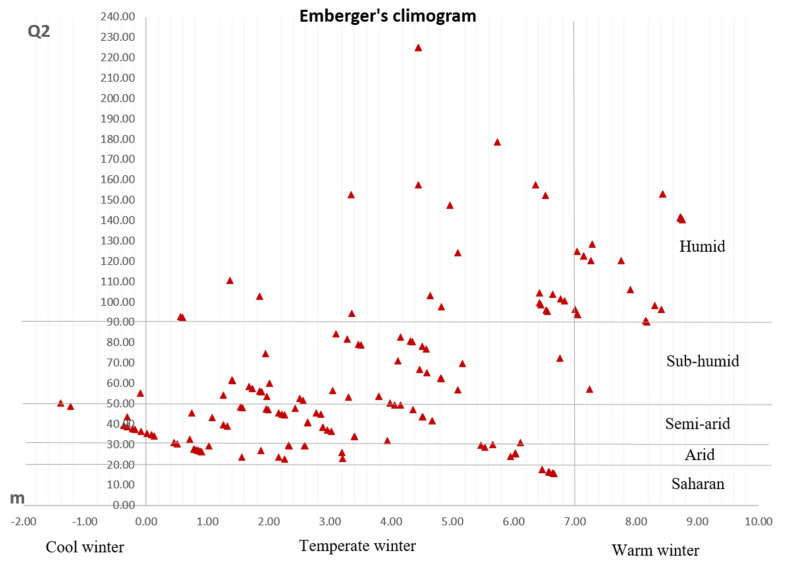
Climogram showing the distribution of the collected wild olive samples according to the Emberger’s coefficient.

The bioclimatic data and the pluviothermic Emberger quotients (Q2) confirmed a wide variability of growing conditions for the 175 collected wild olive samples. These were found to belong to five bioclimatic groups as follows: 61 samples to the humid and temperate/warm winter area, 37 samples to the sub-humid/temperate winter area, 49 samples to the semi-arid/cool-temperate winter area, 24 samples to the arid/temperate winter area, and 4 samples to the Saharan area (Figure 2).

### 3.2. Quantitative Traits Analysis

The results of the descriptive statistics for the 175 samples on the 12 analyzed quantitative variables are summarized in Table 2. The coefficient of variation (CV) indicated a high variability for all descriptors among the wild olive samples, with the highest values observed for the traits “fruit weight” and “stone weight”, and the lowest observed for “stone width”.

A highly positive correlation was observed between fruit and stone traits. In particular, the highest values were recorded between fruit weight/fruit width (r = 0.90), fruit weight/fruit length (r = 0.87), and stone length/fruit length (r = 0.89). On the contrary, very weak correlations were found among all the leaf traits and with the other analyzed traits. (Table 3).

ANOVA along with the pairwise post-hoc Tukey test performed on quantitative traits showed that only the Saharan samples were significantly different (*p* < 0.05) for traits “leaf blade length” and “width”, and for “fruit width” (Table 4).

### 3.3. Qualitative Traits

All qualitative traits were shown to be highly polymorphic except Nipple (NI), Lenticels (LE), and Dimension of Lenticels (DLE) which were monomorphic and were therefore excluded from further analyses. Shannon information index (Hj) was >0.6 for all traits except for the stone number of grooves (Hj = 0.37), reaching the maximum value of 1.28 for the longitudinal curvature of the blade (LCB) which also expressed the highest value of discriminating power (Dj = 0.70, average = 0.54) (Table 5, Supplementary Figure S2).

**Figure 3:**
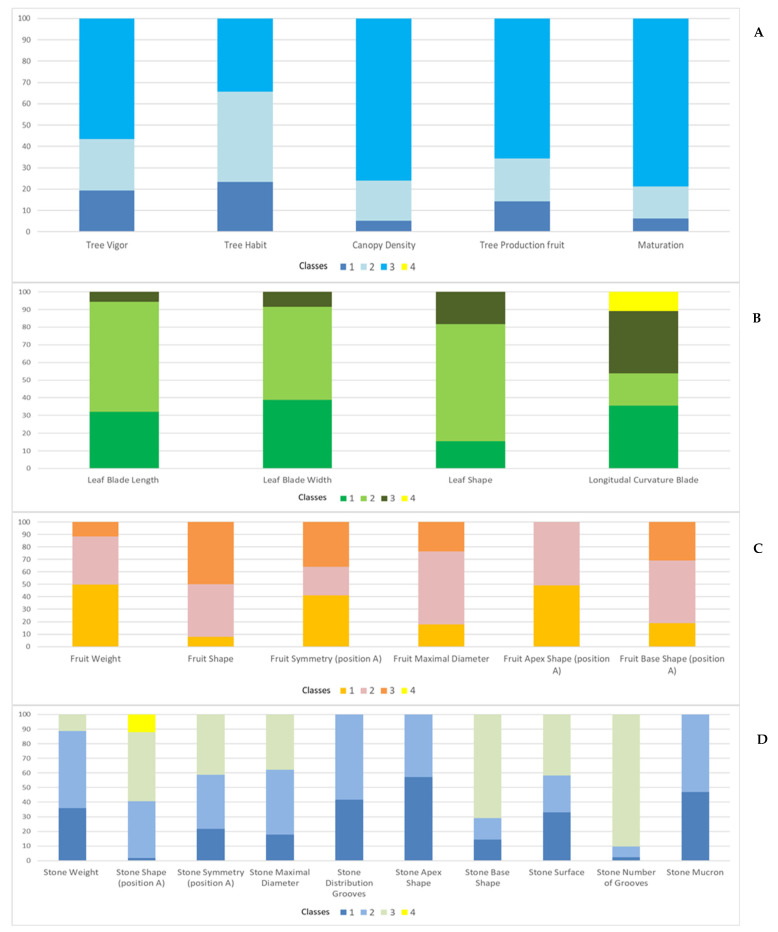
Histograms represent the distribution of the qualitative traits relative to tree habit (**A**), leaf (**B**), fruit (**C**), and stone (**D**) among the analyzed wild olive samples. For each trait (bar), the % of samples falling into each of the four classes considered for descriptors is indicated.

According to the 4 classes considered for qualitative descriptors, wild olive samples were classified as illustrated in Figure 3. Most of the samples were characterized by strong vigor (class 3), spread or erect branches (class 2), compact canopy density (class 3), and high production (class 3) with late maturation (class 3). Leaves were typically elliptic-lanceolate (class 2), with medium or narrow blade width (class 2), medium blade length (class 2), and epinastic (class 1)/hyponastic (class 3) longitudinal curvature blade (Figure 3). Most samples bore fruits of low weight (class 1), oval or long shape (classes 2 and 3), and both symmetrical and asymmetrical (classes 1, 3). They were also characterized by a central maximum diameter (class 2), apex shape rounded or pointed (classes 1, 2), and a fruit base shape usually truncate or pointed (classes 2, 3). The stones were characterized by low or medium weight (classes 1, 2), elliptic shape (class 3), slightly asymmetrical/asymmetrical shape (classes 2, 3), central or toward apex maximal diameter, high number and irregular distribution of grooves, pointed apex and base, a surface scabrous or smooth, and with or without mucron (Figure 3). 

**Figure 4:**
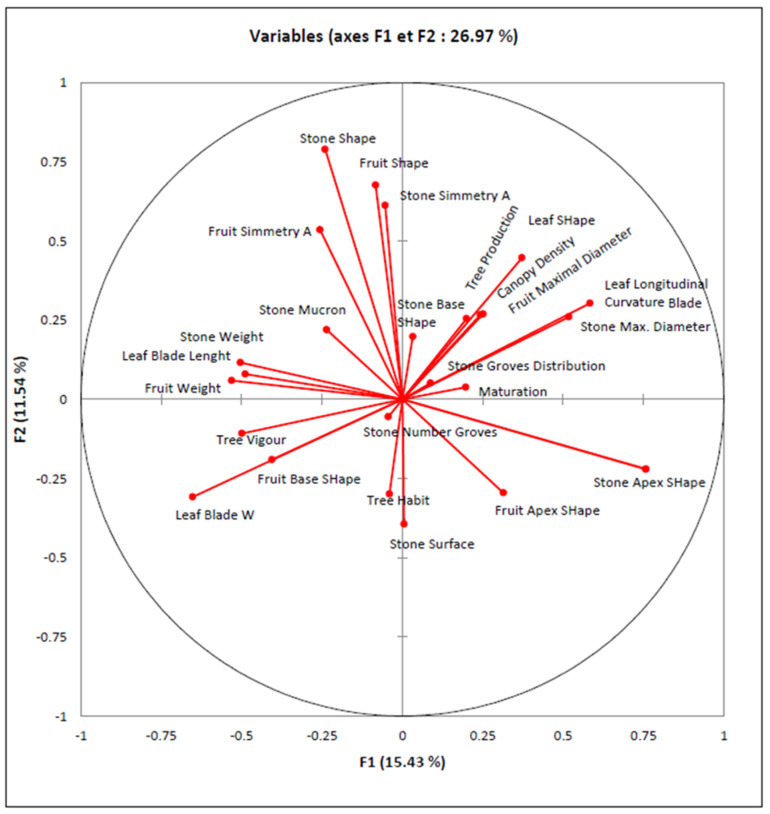
Plot illustrating the relationships among the 25 polymorphic qualitative morphological traits assessed via PCA analysis of 175 wild olive trees (Nipple, Lenticels, and Dimension Lenticels were excluded because they showed to be monomorphic among samples).

The PCA performed on qualitative traits showed that the first two principal components explained 26.97% of the total variance (Figure 4). The first component, PC1, accounted for 15.43%, with the highest contribution from the “stone and fruit weight”, the “stone apex shape”, the “stone maximal diameter”, and the leaf traits “blade width”, “blade length”, and “Longitudinal Curvature Blade”. The second component, PC2, accounted for the 11.54 variance among samples, with the highest contribution from the “fruit and stone shape” and “symmetry” (Figure 4).

### 3.4. Cluster Analysis

**Figure 5:**
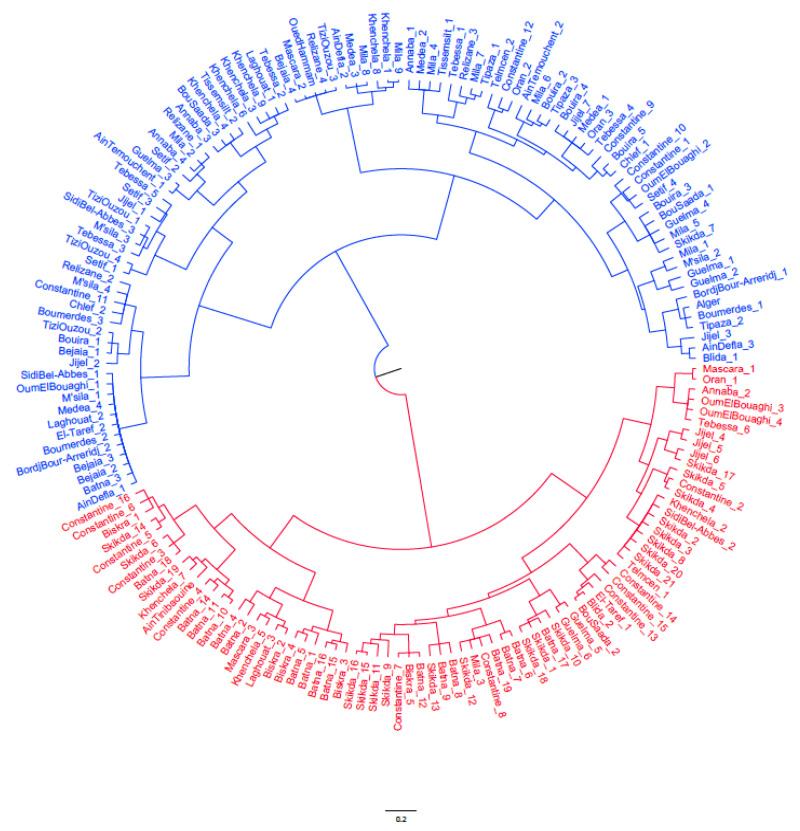
Dendrogram generated by Ward’s method based on morphological markers, illustrating the similarity among the 175 wild olive Algerian samples. The colors red and blue were used to distinguish between the two obtained clusters, Cluster 1 and Cluster 2.

The hierarchical cluster analysis performed on all 32 traits highlighted the relationships between the analyzed wild olive samples, dividing the samples into two clusters, which comprised 99 samples (CI) and 76 samples (C II), respectively (Figure 5). 

**Figure 6:**
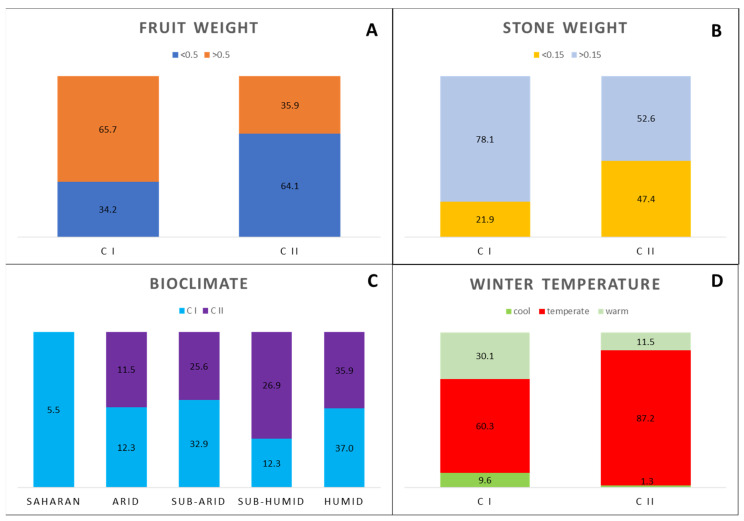
Histogram illustrating the % of olive samples included in clusters I and cluster II of the dendrogram, considering the traits fruit (**A**) and stone (**B**), and the climatic factors (**C**,**D**).

The clustering appeared to be based on fruit and stone traits, with Cluster I including mostly the samples with fruit weight > 0.5 g and medium/large stone (>0.15), and Cluster II comprising mostly the samples with fruit weight < 0.5 and medium/small stone weight (<0.15 g) (Figure 6). On the contrary, no relationship was found with the ecogeographic origin of samples, as Cluster I included samples collected from all the different climatic areas considered, from Saharan to humid, while Cluster II grouped prevalently the samples collected in sub-humid/humid areas, characterized by temperate/warm winters (Figure 6).

## 4. Discussion

In recent years, the loss of genetic variability due to the spread of intensive agriculture and as a result of climate change, has triggered greater attention to the recovery and conservation of wild germplasm. This can be a source of genetic variability useful for overcoming new challenges, such as the recent outbreak of the devastating uncontrolled epidemic of Olive Quick Decline Syndrome (OQDS) caused by the bacterium *Xylella fastidiosa* spp. *pauca* [41]. Mining the available genetic resources of wild olive can allow finding interesting traits to be used in projects for favorable alleles introgression into cultivated olive to face biotic and abiotic stresses [42,43].

Due to its peculiar ecogeographic characteristics, Algeria has vast territories still untouched by anthropization, where wild olive trees still survive in extreme ecoclimatic conditions [24,44]. In recent years, the variability in the Algerian wild olive trees has aroused growing interest, prompting the exploration of the genetic diversity of the species, even in comparison with domestic olive [24,45,46].

The present study enabled the collection of 175 wild olive samples from 34 Algerian provinces located in the north of the country. The specimens were found in five different bioclimatic areas, classified according to the Emberger’s quotients, ranging from the humid areas near the coasts to inland regions in the Saharan Region, confirming the great adaptability of this species to very different climatic environments, including extremes. 

The morphological characterization confirmed a high variability between samples for all observed traits except for Nipple (NI), Lenticels (LE), and Dimension of Lenticels (DLE), which were shown to be monomorphic. The greatest variation was found for the weight of fruit and stone, which reached values higher than those already reported [35]. Fruit and stone descriptors showed a strong correlation and were very effective in discriminating between olive genotypes. On the contrary, leaf traits exhibited little variation and discrimination power, confirming previous reports for both wild olive [35] and the cultivated one [24,47]. In particular, the endocarp traits proved to be very stable and highly discriminating compared to other traits more influenced by environmental conditions [48,49]. In fact, they are frequently used to catalog olive cultivars because they can be effectively kept for a long time and are easily exchangeable [50,51]. 

The mean fruit and stone weight for the wild olive samples was 0.61 g and 0.19 g, respectively, reflecting values consistent with those previously reported for the wild olive [24,35,52,53,54]. However, several samples showed a medium-large fruit size, more similar to that of the cultivated olive tree. One explanation for this could be that these samples are not true wild olives but, more likely, feral forms derived from a hybridization event with cultivated olives or cultivars that have escaped cultivation. 

In addition, fruit and stone size were at the base of the results of the clustering analysis, which included the samples with larger fruits and seeds in Cluster I, and the samples with smaller fruits and seeds in Cluster II. On the contrary, the dendrogram did not show any link with the ecoclimatic origin of samples, as both clusters included samples from all climatic areas, although Cluster II collected more samples from sub-humid/humid areas. This is in contrast to previous observations obtained on the same Algerian wild olive trees through a genetic analysis with 16 microsatellite markers, which grouped the samples according to the growing climatic conditions [25]. This result could be due to the physical isolation of trees, which restricts gene flow between populations, leading to a marked genetic differentiation which, instead, does not correspond to a clear morphological variation. Only the Saharan samples resulted in significantly different leaf traits and fruit, indicating the potential of the wild trees belonging to this area and the necessity to deeply characterize them.

Overall, this study allowed us to collect and morphologically characterize a very diverse heritage of wild olive growing in Algeria. This germplasm showed valuable variability for many traits, and the Saharan samples, in particular, appeared to be significantly different for leaf traits, from samples growing in other bioclimatic areas. Deeper studies are needed, using more descriptors and integrating the morphological data with genetic studies, to better understand the impact the bioclimatic variables have on wild olive diversity. This will allow us to effectively exploit the potential of the wild olive, whose variation deserves to be adequately cataloged and preserved for future exploitation in breeding programs. 

## Figures and Tables

**Table 1:** Qualitative and quantitative descriptors were used for the characterization of Algerian wild olive samples, following the UPOV methodology. Eight traits were considered as both quantitative and qualitative descriptors.

Organs	Trait	Trait Code	Classes			
TREE ARCHITECTURE	Tree Vigor	TV ^1^	1 = Weak	2 = Medium	3 = Strong	
	Tree Habit	TH ^1^	1 = Falling down	2 = Spread out	3 = Erected	
	Canopy Density	CD ^1^	1 = Loose	2 = Medium	3 = Compact	
	Tree Production fruit	TP ^1^	1 = Weak	2 = Medium	3 = Strong	
	Maturation	M ^1^	1 = Early	2 = Medium	3 = Late	
LEAF SHAPE AND SIZE	Shape	LSH ^3^	1 = Elliptic (Length/Width < 4)	2 = Elliptic-lanceolate (Length/Width 4–6)	3 = Lanceolate (Length/Width > 6)	
	Blade Length (cm)	LBL ^3^	1 = Short (<5 cm)	2 = Medium (5–7 cm)	3 = Long (>7 cm)	
	Blade Width (cm)	LBW ^3^	1 = Narrow (<1 cm)	2 = Medium (1–1.5 cm)	3 = Wide (>1.50 cm)	
	Longitudinal Curvature Blade	LCB ^1^	1 = Epinastic	2 = Flat	3 = Hyponastic	4 = Helicoidal
FRUIT SHAPE AND SIZE	Weight (g)	FW ^3^	1 = Low (<0.5 g)	2 = Medium (0.5–1 g)	3 = High (>1 g)	
	Shape	FSH ^3^	1 = Spherical (L/A < 1.25)	2 = Oval (Length/Width 1.25–1.45)	3 = Longer (Length/Width > 1.45)	
	Symmetry (position A)	FSA ^1^	1 = Symmetrical	2 = Slightly Asymmetrical	3 = Asymmetrical	
	Maximal Diameter	FMD ^1^	1 = Toward base	2 = Central	3 = Toward apex	
	Apex Shape (position A)	FASH ^1^	1 = Pointed	2 = Rounded		
	Base Shape (position A)	FBSH ^1^	1 = Rounded	2 = Truncate	3 = Pointed	
	Nipple	NI ^1^	1 = Absent	2 = Tenuous		
	Lenticels	LE ^1^	1 = Few	2 = Many		
	Dimension Lenticels	DLE ^1^	1 = Small	2 = Large		
	Length (cm)	FL ^2^	/	/	/	
	Width (cm)	FWI ^2^	/	/	/	
STONE SHAPE AND SIZE	Weight (g)	SW ^3^	1 = Low (<0.15 g)	2 = Medium (0.15–0.30 g)	3 = High (>0.3 g)	
	Shape (position A)	SSH ^3^	1 = Spherical (Length/Width < 1.4)	2 = Oval (Length/Width 1.4–1.8)	3 = Elliptic (Length/Width 1.8–2.2)	4 = Longer (Length/Width > 2.2)
	Symmetry (position A)	SSA ^1^	1 = Symmetrical	2 = Slightly Asymmetrical	3 = Asymmetrical	
	Maximal Diameter	SMD ^1^	1 = Toward base	2 = Central	3 = Toward apex	
	Groves distribution	SDG ^1^	1 = Regular	2 = Irregular		
	Apex Shape	SASH ^1^	1 = Pointed	2 = Rounded		
	Base Shape	SBSH ^1^	1 = Rounded	2 = Truncate	3 = Pointed	
	Surface	SS ^1^	1 = Smooth	2 = Rugose	3 = Scabrous	
	Number of Grooves	SNG ^3^	1 = Reduced (<6)	2 = Medium (6–7)	3 = High (>7)	
	Mucron	SM ^1^	1 = Without mucron	2 = With mucron		
	Length (cm)	SL ^2^	/	/	/	
	Width (cm)	SWI ^2^	/	/	/	

^1^ qualitative trait; ^2^ quantitative trait; ^3^ trait considered as both qualitative and quantitative.

**Table 2:** Results for descriptive statistics obtained for the 175 samples based on the 12 analyzed quantitative traits.

Trait	Range	Mean	Standard Deviation	CV (%)
Leaf Blade Length (cm)	2.97–8.99	5.38	0.96	17.87%
Leaf Blade Width (cm)	0.56–2.05	1.11	0.29	26.19%
Leaf Shape (L/I)	2.87–9.31	5.14	1.24	24.09%
Fruit Weight (g)	0.11–1.87	0.61	0.35	57.82%
Fruit Length (cm)	0.64–2.19	1.22	0.24	19.81%
Fruit Width (cm)	0.48–1.36	0.84	0.16	19.52%
Fruit Shape (L/I)	1.09–2.13	1.48	0.18	11.86%
Stone Weight (g)	0.04–0.98	0.19	0.10	51.57%
Stone Length (cm)	0.56–1.64	1.04	0.18	17.16%
Stone Width (cm)	0.38–0.81	0.56	0.07	11.70%
Stone Shape (L/I)	1.32–2.93	1.87	0.26	13.93%
Stone Number of Grooves (SNG)	4.18–14.65	9.57	1.89	19.73%

**Table 3:** Correlation between 12 morphological quantitative traits based on individual tree averages. The values higher than 0.7 indicate a strong correlation and are reported in bold.

Descriptors	LBW	LSH	FW	FL	FWI	FSH	SW	SL	SWl	SSH	SNG
Leaf Blade Length (cm) (LBL)	0.57 ***	0.15	0.41 ***	0.45 ***	0.42 ***	0.08	0.32 ***	0.35 ***	0.22 **	0.25 ***	0.00
Leaf Blade Width (cm) (LBW)		−0.67 ***	0.36 ***	0.37 ***	0.45 ***	−0.14	0.33 ***	0.18 *	0.26 ***	0.01	−0.10
Leaf Shape (L/I) (LSH)			−0.10	−0.07	−0.20 **	0.27 ***	−0.11	0.08	−0.13	0.21 **	0.13
Fruit Weight (g) (FW)				0.87 ***	0.90 ***	−0.03	0.72 ***	0.69 ***	0.71 ***	0.26 ***	0.05
Fruit Length (cm) (FL)					0.86 ***	0.30 ***	0.72 ***	0.89 ***	0.69 ***	0.55 ***	0.08
Fruit Width (cm) (FWI)						−0.22 **	0.71 ***	0.60 ***	0.76 ***	0.12	0.03
Fruit Shape (L/I) (FSH)							0.03	0.56 ***	−0.11	0.85 ***	0.11
Stone Weight (g) (SW)								0.64 ***	0.75 ***	0.17	0.14
Stone Length (cm) (SL)									0.66 ***	0.74 ***	0.09
Stone Width (cm) (SW)										0.00	0.04
Stone Shape (L/I) (SSH)											0.07

*** significant at *p* < 0.001; ** significant at *p* < 0.01; * significant at *p* < 0.05.

**Table 4:** Descriptive statistics with average ± standard deviation for each of the 12 quantitative morphological traits in each bioclimatic area. Different letters indicate significant difference (*p* < 0.05).

	Trait											
Bioclimate	Leaf Blade Length (cm)	Leaf Blade Width (cm)	Leaf Shape (L/I)	Fruit Weight (g)	Fruit Length (cm)	Fruit Width (cm)	Fruit Shape (L/I)	Stone Weight (g)	Stone Length (cm)	Stone Width (cm)	Stone Shape (L/I)	Stone Number of Grooves
Sub-humid	5.25 ± 0.83 a	1.09 ± 0.26 a	5.13 ± 1.37 a,b	0.59 ± 0.31 a	1.22 ± 0.21 a	0.85 ± 0.16 a	1.46 ± 0.17 a,b	0.22 ± 0.16 a	1.05 ± 0.17 a	0.58 ± 0.07 a	1.84 ± 0.23 a	9.71 ± 2.08 a
Humid	5.26 ± 0.87 a	1.09 ± 0.30 a	5.12 ± 1.38 a,b	0.61 ± 0.34 a	1.20 ± 0.22 a	0.83 ± 0.16 a	1.47 ± 0.17 a,b	0.18 ± 0.07 a	1.01 ± 0.16 a	0.54 ± 0.06 a	1.87 ± 0.25 a	9.55 ± 1.71 a
Semi-arid	5.56 ± 1.10 a	1.16 ± 0.27 a	4.93 ± 0.99 a,b	0.57 ± 0.35 a	0.57 ± 0.26 a	0.81 ± 0.15 a	1.48 ± 0.17 a,b	0.18 ± 0.08 a	1.04 ± 0.20 a	0.56 ± 0.07 a	1.86 ± 0.30 a	9.42 ± 2.16 a
Arid	5.34 ± 0.91 a	0.99 ± 0.28 a	5.74 ± 1.06 a	0.69 ± 0.45 a	1.30 ± 0.30 a	0.85 ± 0.20 a	1.55 ± 0.20 a	0.19 ± 0.07 a	1.10 ± 0.18 a	0.55 ± 0.06 a	1.98 ± 0.26 a	9.77 ± 1.59 a
Saharan	6.64 ± 1.25 b	1.56 ± 0.27 b	4.49 ± 0.48 b	0.89 ± 0.28 a	1.41 ± 0.17 a	1.04 ± 0.12 b	1.37 ± 0.19 b	0.25 ± 0.09 a	1.05 ± 0.21 a	0.60 ± 0.05 a	1.74 ± 0.21 a	9.11 ± 1.38 a

**Table 5:** Results obtained for diversity indices on qualitative traits analysis: number of total classes (KT), number of observed classes (Kj), Shannon’s information index (Hj) and Discriminating power (Dj) are reported.

Trait	Trait Code	KT	Kj	Hj	Dj
Tree Vigor	TV	3	3	0.979	0.582
Tree Habit	TH	3	3	1.071	0.649
Canopy Density	CD	3	3	0.682	0.390
Tree Production fruit	TP	3	3	0.881	0.511
Maturation	M	3	3	0.635	0.346
Leaf Shape	LSH	3	3	0.868	0.501
Leaf Blade Length (cm)	LBL	3	3	0.841	0.513
Leaf Blade Width (cm)	LBW	3	3	0.920	0.566
Longitudinal Curvature Blade	LCB	4	4	1.281	0.701
Fruit Weight (g)	FW	3	3	0.988	0.601
Fruit Shape	FSH	3	3	0.910	0.566
Fruit Symmetry (position A)	FSA	3	3	1.066	0.646
Fruit Maximal Diameter	FMD	3	3	0.956	0.565
Fruit Apex Shape (position A)	FASH	2	2	0.693	0.500
Fruit Base Shape (position A)	FBSH	3	3	1.021	0.614
Nipple	NI	2	1	-	-
Lenticels	LE	2	1	-	-
Dimension Lenticels	DLE	2	1	-	-
Stone Weight (g)	SW	3	3	0.979	0.594
Stone Shape (position A)	SSH	4	4	1.046	0.609
Stone Symmetry (position A)	SSA	3	3	1.063	0.645
Stone Maximal Diameter	SMD	3	3	1.034	0.627
Stone Distribution Grooves	SDG	2	2	0.682	0.489
Stone Apex Shape	SASH	2	2	0.680	0.487
Stone Base Shape	SBSH	3	3	0.795	0.448
Stone Surface	SS	3	3	1.081	0.655
Stone Number of Grooves	SNG	3	3	0.366	0.175
Stone Mucron	SM	2	2	0.691	0.498
Average				0.89	0.54
Min				0.37	0.18
Max				1.28	0.70

## Data Availability

The data presented in this study are available on request from the corresponding author.

## Supplementary Materials

The following supporting information can be downloaded at: https://www.mdpi.com/article/10.3390/plants11141803/s1, Supplementary Table S1: List of the 175 samples analyzed in this study; Supplementary Figure S1: An example of some descriptors indicated by UPOV for morphological characterization of olive; Supplementary Figure S2: Pictures representing some of the qualitative traits of the samples analyzed in this study.

## References

[1] Jukić Špika M., Liber Z., Montemurro C., Miazzi M.M., Ljubenkov I., Soldo B., Žanetić M., Vitanović E., Politeo O., Škevin D. (2022). Quantitatively Unraveling Hierarchy of Factors Impacting Virgin Olive Oil Phenolic Profile and Oxidative Stability. Antioxidants.

[2] Green P.S. (2002). A revision of *Olea* L. (*Oleaceae*). Kew Bull..

[3] Besnard G., Rubio de Casas R. (2016). Single vs. multiple independent olive domestications: The jury is (still) out. New Phytol..

[4] Terral J.F., Arnold-Simard G. (1996). Beginnings of olive cultivation in eastern Spain in relation to Holocene bioclimatic changes. Quat. Res..

[5] Baldoni L., Tosti N., Ricciolini C., Belaj A., Arcioni S., Pannelli G., Germanà M.A., Mulas M., Porceddu A. (2006). Genetic structure of wild and cultivated olives in the Central Mediterranean Basin. Ann. Bot..

[6] Klepo T., De la Rosa R., Satovic Z., León L., Belaj A. (2013). Utility of wild germplasm in olive breeding. Sci. Hortic..

[7] Besnard G., Khadari B., Navascués M., Fernández-Mazuecos M., El Bakkali A., Arrigo N., Baali-Cherif D., De Caraffa V.B.-B., Santoni S., Vargas P. (2013). The complex history of the olive tree: From Late Quaternary diversification of Mediterranean lineages to primary domestication in the northern Levant. Proc. R. Soc. B Biol. Sci..

[8] Aranda S., Montes-Borrego M., Jiménez-Díaz R.M., Landa B.B. (2011). Microbial communities associated with the root system of wild olives (*Olea europaea* L. subsp. *europaea* var. *sylvestris*) are good reservoirs of bacteria with antagonistic potential against *Verticillium dahliae*. Plant Soil.

[9] Arias-Calderón R., Rodríguez-Jurado D., León L., Bajarano-Alcázar J., De la Rosa R., Belaj A. (2015). Pre-breeding for resistance to *Verticillium* wilt in olive: Fishing in the wild relative gene pool. Crop Prot..

[10] Díaz-Rueda P., Franco-Navarro J., Messora R., Espartero J., Rivero-Núñez C., Aleza P., Capote N., Cantos M., García-Fernández J.L., de Cires A. (2020). SILVOLIVE, a germplasm collection of wild subspecies with high genetic variability as a source of rootstocks and resistance genes for olive breeding. Front. Plant Sci..

[11] Hannachi H., Nasri N., Elfalleh W., Tlili N., Ferchichi A., Msallem M. (2013). Fatty acids, sterols, polyphenols and chlorophylls of olive oils obtained from Tunisian wild olive trees (*Olea europaea* L. var. sylvestris). Int. J. Food Prop..

[12] Baccouri B., Guerfel M., Zarrouk W., Taamalli W., Daoud D., Zarrouk M. (2011). Wild olive (*Olea europaea* L.) selection for quality oil production. J. Food Biochem..

[13] Dabbou S., Dabbou S., Selvaggini R., Urbani S., Taticchi A., Servili M., Hammami M. (2011). Comparison of the chemical composition and the organoleptic profile of virgin olive oil from two wild and two cultivated Tunisian Olea Europaea. Chem. Biodivers..

[14] Baali-Cherif D., Besnard G. (2005). High Genetic Diversity and Clonal Growth in Relict Populations of *Olea europaea* subsp. *laperrinei* (*Oleaceae*) from Hoggar, Algeria. Ann. Bot..

[15] Besnard G., Anthelme F., Baali-Cherif D. (2012). The Laperrine’s olive tree (*Oleaceae*): A wild genetic resource of the cultivated olive and a model-species for studying the biogeography of the Saharan Mountains. Acta Bot. Gall..

[16] https://www.fao.org/faostat/en/#data/QCL/visualize.

[17] Mendil M., Sebai A. (2006). Catalogue des Variétés Algérienne de L’olivier: L’olivier en Algérie.

[18] Boucheffa S., Miazzi M.M., di Rienzo V., Mangini G., Fanelli V., Tamendjari A., Pignone D., Montemurro C. (2017). The coexistence of oleaster and traditional varieties affects genetic diversity and population structure in Algerian olive (*Olea europaea*) germplasm. Genet. Resour. Crop Evol..

[19] Di Rienzo V., Sion S., Taranto F., D’Agostino N., Montemurro C., Fanelli V., Sabetta W., Boucheffa S., Tamendjari A., Pasqualone A. (2018). Genetic flow among olive populations within the Mediterranean basin di Rienzo. PeerJ.

[20] Di Rienzo V., Miazzi M.M., Fanelli V., Sabetta W., Montemurro C. (2018). The preservation and characterization of Apulian olive germplasm biodiversity. Acta Hortic..

[21] Saddoud Debbabi O.S., Miazzi M.M., Elloumi O., Fendri M., Ben Amar F., Savoia M., Sion S., Souabni H., Mnasri S.R., Ben Abdelaali S. (2020). Recovery, assessment, and molecular characterization of minor olive genotypes in Tunisia. Plants.

[22] Saddoud Deddabi O., Montemurro C., Ben Maachia S., Ben Amar F., Fanelli V., Gadaleta S., El Riachy M., Chehade A., Siblini M., Boucheffa S. (2020). A hot spot of olive biodiversity in the Tunisian Oasis of Degache. Diversity.

[23] Miazzi M.M., di Rienzo V., Mascio I., Montemurro C., Sion S., Sabetta W., Vivaldi G.A., Camposeo S., Caponio F., Squeo G. (2020). Ger.OP: An Integrated Project for the Recovery of Ancient and Rare Olive Germplasm. Front. Plant Sci..

[24] Boucheffa S., Tamendjari A., Sanchez-Gimeno A.C., Rovellini P., Venturini S., di Rienzo V., Miazzi M.M., Montemurro C. (2019). Diversity assessment of Algerian wild and cultivated olives (*Olea europaea* L.) by molecular, morphological, and chemical traits. Eur. J. Lipid Sci. Technol..

[25] Falek W., Sion S., Montemurro C., Mascio I., Gadaleta S., Fanelli V., Savoia M.A., Piarulli L., Bechkri S., Khelifi D. (2022). Molecular diversity and ecogeographic distribution of Algerian wild olives (*Olea europaea* subsp. *europaea* var. *sylvestris*). Sci. Agric..

[26] D’Agostino N., Taranto F., Camposeo S., Mangini G., Fanelli V., Gadaleta S., Miazzi M.M., Pavan S., di Rienzo V., Sabetta W. (2018). GBS-derived SNP catalogue unveiled wide genetic variability and geographical relationships of Italian olive cultivars. Sci. Rep..

[27] Taranto F., D’Agostino N., Pavan S., Fanelli V., Di Rienzo V., Sabetta W., Miazzi M.M., Zelasco S., Perri E., Montemurro C. (2016). Single nucleotide polymorphism (SNP) diversity in an olive germplasm collection. VIII Int. Olive Symp..

[28] Sion S., Savoia M.A., Gadaleta S., Piarulli L., Mascio I., Fanelli V., Montemurro C., Miazzi M.M. (2021). How to choose a good marker to analyze the olive germplasm (*Olea europaea* L.) and derived products. Genes.

[29] Unver T., Wu Z., Sterck L., Turktas M., Lohaus R., Li Z., Yang M., He L., Deng T., Escalante F.J. (2017). Wild olive genome and oil biosynthesis. Proc. Natl. Acad. Sci. USA.

[30] Boukhari R., Ameur A.A., Innal H., Gaouar S.B.S. (2020). First morphological characterization of autochthonous olive (*Olea europaea* L.) denominations from central and eastern of Algeria. Acta Agric. Slov..

[31] Khouatmiani K., Belhadj S., Tonetto A., Assie A., Mevy J.P., Gauquelin T. (2021). Variability of eight Algerian oleaster ecotypes (*Olea europaea* subsp. *europaea* var. *sylvestris* [Mill.] Lehr): Pollen and exine morphology in relation to geo-climatic effect. Grana.

[32] UPOV (1985). Guidelines for the Conduct of Tests for Distinctness, Homogeneity and Stability. www.upov.int.

[33] Bechkri S., Khelifi D. (2017). Variation in *Vicia sativa* s.l. from Algeria based on morphological characters and ecogeographic parameters. Genet. Resour. Crop Evol..

[34] Emberger L. (1955). Une classification bio-géographique des climats. Rech. Trav. Lab. Bot. Fac. Sci. Montp. Série Bot..

[35] Belaj A., León L., Satovic Z., De la Rosa R. (2011). Variability of wild olives (*Olea europaea* subsp. *europaea* var. *sylvestris*) analyzed by agro—Morphological traits and SSR markers. Sci. Hortic..

[36] Sokal R.R., Braumann C.A. (1980). Significance tests for coefficients of variation and variability profiles. Syst. Zool..

[37] Peakall R., Smouse P.E. (2012). GenALEx 6.5, genetic analysis in Excel. Population genetic software for teaching and research. Bioinformatics.

[38] Tessier C., David J., This P., Boursiquot J.M., Charrier A. (1999). Optimization of the choice of molecular markers for varietal identification in *Vitis vinifera* L.. Theor. Appl. Genet..

[39] Jolliffe I.T., Cadima J. (2016). Principal component analysis: A review and recent developments. Phil. Trans. R. Soc. A.

[40] Felsenstein J. (1985). Confidence limits on phylogenies: An approach using the bootstrap. Evolution.

[41] Saponari M., Boscia D., Altamura G., Loconsole G., Zicca S., D’Attoma G., Morelli M., Palmisano F., Saponari A., Tavano D. (2017). Isolation and pathogenicity of *Xylella fastidiosa* associated to the olive quick decline syndrome in southern Italy. Sci. Rep..

[42] Fanelli V., Mascio I., Falek W., Miazzi M.M., Montemurro C. (2022). Current Status of Biodiversity Assessment and Conservation of Wild Olive (*Olea europaea* L. subsp. *europaea* var. *sylvestris*). Plants.

[43] Sion S., Taranto F., Montemurro C., Mangini G., Camposeo S., Falco V., Gallo A., Mita G., Saddoud Debbabi O., Ben Amar F. (2019). Genetic characterization of Apulian olive germplasm as potential source in new breeding programs. Plants.

[44] Besnard G., Baradat P., Bervillé A. (2001). Genetic relationships in the olive (*Olea europaea* L.) reflect multilocal selection of cultivars. Theor. Appl. Genet..

[45] Breton C., Tersac M., Bervillé A. (2006). Genetic diversity and gene flow between the wild olive (oleaster, *Olea europaea* L.) and the olive: Several Plio-Pleistocene refuge zones in the Mediterranean basin suggested by simple sequence repeats analysis. J. Biogeogr..

[46] Kassa A., Konrad H., Geburek T. (2019). Molecular diversity and gene flow within and among different subspecies of the wild olive (*Olea europaea* L.): A review. Flora.

[47] Sorkheh K., Khaleghi E. (2016). Molecular characterization of genetic variability and structure of olive (*Olea europaea* L.) germplasm collection analyzed by agromorphological traits and microsatellite markers. Turk. J. Agric. For..

[48] Koubouris G.C., Avramidou E., Metzidakis I.T., Petrakis P.V., Sergentani C.K., Doulis A.G. (2019). Phylogenetic and evolutionary applications of analyzing endocarp morphological characters by classification binary tree and leaves by SSR markers for the characterization of olive germplasm. Tree Genet. Genomes.

[49] Fendri M., Trujillo I., Trigui A., Rodríguez-García M., Ramírez J.D. (2010). Simple Sequence Repeat Identification and Endocarp Characterization of Olive Tree Accessions in a Tunisian Germplasm Collection. HortScience.

[50] D’Imperio M., Viscosi V., Scarano M.T., D’Andrea M., Zullo B.A., Pilla F. (2011). Integration between molecular and morphological markers for the exploitation of olive germoplasm (*Olea europaea*). Sci. Hortic..

[51] Trujillo I., Ojeda M.A., Urdiroz N.M., Potter D., Barranco D., Rallo L., Diez C.M. (2013). Identification of the worldwide olive germplasm bank of Córdoba (Spain) using SSR and morphological markers. Tree Genet. Genomes.

[52] Hannachi H., Breton C., Msallem M., El Hadj S.B., El Gazzah M., Bervillé A. (2008). Differences between native and introduced olive cultivars as revealed by morphology of drupes, oil composition and SSR polymorphism: A case study in Tunisia. Sci. Hort..

[53] Rodrigues N., Pinho T., Casal S., Peres A.M., Baptista P., Pereira J.A. (2020). Chemical Characterization of Oleaster, *Olea europaea* var. *sylvestris* (Mill.) Lehr., Oils from Different Locations of Northeast Portugal. Appl. Sci..

[54] Hannachi H., Gómez J.J.M., Saadaoui E., Cervantes E. (2017). Stone diversity in wild and cultivated olive trees (*Olea europaea* L.). Dendrobiology.

