# Supplementary material for: Morphological and Eco-Geographic Variation in Algerian Wild Olives
Source: Plants (Basel). 2022 Jul 8;11(14):1803. doi: 10.3390/plants11141803 (PMC9319472; doi:10.3390/plants11141803)
Supplement: Supplementary file 1 [file plants-11-01803-s001.zip › plants-1735974-supplementary.pdf]

Article

Morphological and eco-geographic variation in Algerian wild olives

Wahiba Falek [1], Isabella Mascio [2], Susanna Gadaleta [3], Valentina Fanelli [2], Sakina Bechkri [1], Douadi Khelifi [1], Monica Marilena Miazzi [2,*] and Cinzia Montemurro [2,3]

[1] Ecole Nationale Supérieure de Biotechnologie, 251000, Constantine, Algeria;
[2] Department of Soil, Plant and Food Sciences, University of Bari Aldo Moro, 70125, Bari, Italy;
[3] Spin off Sinagri s.r.l., University of Bari Aldo Moro, 70125, Bari, Italy

* Correspondence: monicamarilena.miazzi@uniba.it (M.M.M.)

Wahiba Falek and Isabella Mascio should be considered joint first author

Supplementary Material

Supplementary Table S1. List of the 175 samples analyzed in this study. For each sample, code, origin and the following ecogeographic parameters are reported: altitude expressed in meter, latitude, longitude, minimum temperature of the coldest month (m °C), Emberger coefficient (Q), bioclimate and winter.

Code	Origin	Altitude(m)	Latitude	Longitude	m	Q	Bioclimate	Winter
AinDefla_1	Ain Defla, Bir Ouled Khlifa	529	36°01'8.72"N	2°10'2.20"E	4.57	76.83	Sub-humid	Temperate
AinDefla_2	Ain Defla, Djbel Tarak Ben Zied	832	35°58'3.02"N	2°07'0.59"E	3.36	94.47	Humid	Temperate
AinDefla_3	Ain Defla, Djbel Tarak Ben Zied	634	36°0'00.41"N	2°09'0.72"E	4.15	82.82	Sub-humid	Temperate
AinTémouchent_1	Ain Témouchent, Ain El Talba	245	35°17'02.83"N	1°13'2.00"W	6.76	72.36	Sub-humid	Temperate
AinTémouchent_2	Ain Témouchent, Chaabat Ellehem	124	35°21'09.89"N	1°05'7.31"W	7.24	57.26	Sub-humid	Warm winter
AinTinibaouine	Ain Tinibaouine	918	35°34'34.80"N	5°43'46.95E	0.02	35.36	Semi-arid	Temperate
Alger	Alger, Douira	189	36°40'01.39"N	2°59'8.81"E	7.75	120.36	Humid	Warm winter
Annaba_1	Annaba, Ain Berda	73	36°42'4.81"N	7°37'4.32"E	6.42	99.39	Humid	Temperate
Annaba_2	Annaba, Djbel El Boni	40	36°52'0.89"N	7°44'8.51"E	6.55	95.32	Humid	Temperate
Annaba_3	Annaba, Djbel El Boni	46	36°52'0.38"N	7°44'9.69"E	6.53	96.06	Humid	Temperate
Annaba_4	Annaba, Djbel Oued El Aneb	66	36°52'7.25"N	7°27'5.18"E	6.45	98.52	Humid	Temperate
Batna_1	Batna, Ain Touta	942	35°22'34.35"N	5°55'8.21"E	-0.08	36.36	Semi-arid	Cool winter
Batna_2	Batna, Ain Touta Mafa	808	35°17'00.09"N	5°52'5.96"E	0.46	30.85	Semi-arid	Temperate
Batna_3	Batna, Djbel Mafa	1000	35°17'01.03"N	5°52'4.42"E	-0.31	38.78	Semi-arid	Cool winter
Batna_4	Batna, Ghassira	900	35°06'51.25"N	6°7'20.63"E	0.09	34.62	Semi-arid	Temperate
Batna_5	Batna, Kef Fercha	977	35°26'00.25"N	5°42'2.92"E	-0.22	37.82	Semi-arid	Cool winter
Batna_6	Batna, Mafa Egzar Aliou Saîd	890	35°15'06.57"N	5°53'6.60"E	0.13	34.21	Semi-arid	Temperate
Batna_7	Batna, Mafa Egzar Aliou Saîd	1012	35°15'06.57"N	5°53'6.60"E	-0.36	39.29	Semi-arid	Cool winter
Batna_8	Batna, Ouled Aouf	1270	35°26'51.98"N	5°44'56.93E	-1.39	50.38	Sub-humid	Cool winter
Batna_9	Batna, Ouled Si Slimane	794	35°34'43.30"N	5°40'17.00E	0.51	30.28	Semi-arid	Temperate
Batna_10	Batna, Sefiane Ainsoula	726	35°27'05.83"N	5°33'9.23"E	0.78	27.54	Arid	Temperate
Batna_11	Batna, Sefiane Djbelkaf El Saïh	729	35°27'05.83"N	5°33'9.23"E	0.77	27.66	Arid	Temperate
Batna_12	Batna, Sefiane Djbelkaf El Saïh	729	35°27'05.83"N	5°33'9.23"E	0.85	26.90	Arid	Temperate
Batna_13	Batna, Sefiane Djbelkaf El Saïh	729	35°27'05.83"N	5°33'9.23"E	0.85	26.90	Arid	Temperate
Batna_14	Batna, Sefiane koudiaa	701	35°26'05.32"N	5°34'6.00"E	0.88	26.54	Arid	Temperate
Batna_15	Batna, Sefiane Safsafa	717	35°26'09.14"N	5°34'4.70"E	0.82	27.18	Arid	Temperate
Batna_16	Batna, Seggana	696	35°20'49.88"N	5°38'2.48"E	0.90	26.34	Arid	Temperate

Batna_17	Batna, Tafran Ain Tamgalft	967	35°25'09.65''N	5°42'5.63''E	-0.18	37.40	Semi-arid	Cool winter
Batna_18	Batna, Taxlent	1232	35°36'44.41''N	5°48'41.47E	-1.24	48.71	Semi-arid	Cool winter
Béjaia_1	Béjaia, Boulimat	67	36°48'06.75''N	4°59'5.26''E	7.14	122.65	Humid	Warm winter
Béjaia_2	Béjaia, Kseur	94	36°39'09.46''N	4°50'4.79''E	7.03	124.69	Humid	Warm winter
Béjaia_3	Béjaia, Oued Daas	36	36°51'06.65''N	4°48'5.80''E	7.26	120.33	Humid	Warm winter
Béjaia_4	Béjaia, Oued Ghir	67	36°42'04.63''N	4°56'9.61''E	7.14	122.65	Humid	Warm winter
Biskra_1	Biskra, Doucen	191	34°36'54.07''N	5°5'55.55''E	6.46	17.71	Saharien	Temperate
Biskra_2	Biskra, El Ghrous	160	34°42'21.84''N	5°15'49.49E	6.59	16.42	Saharien	Temperate
Biskra_3	Biskra, Foughala	147	34°42'47.89''N	5°19'6.99''E	6.64	15.88	Saharien	Temperate
Biskra_4	Biskra, Lioua	1047	34°37'33.83''N	5°23'07.92E	3.04	56.30	Sub-humid	Temperate
Biskra_5	Biskra, Tolga	142	34°41'44.90''N	5°22'35.00E	6.66	15.67	Saharien	Temperate
Blida_1	Blida, Djbel Chréa	763	36°27'3.16''N	2°52'1.19''E	4.96	147.37	Humid	Temperate
Blida_2	Blida, Djbel Chréa	892	36°27'2.59''N	2°51'6.48''E	4.44	157.65	Humid	Temperate
BordjBour-Arreridj_1	BordjBour-Arreridj, Bni Lalam	731	36°16'03.39''N	4°47'4.15''E	2.59	29.23	Arid	Temperate
BordjBour-Arreridj_2	BordjBour-Arreridj, Djbel El Zbiat	730	36°14'06.48''N	4°49'4.03''E	2.59	29.19	Arid	Temperate
Bouira_1	Bouira, Ait EL Azizz	570	36°25'1.39''N	3°54'2.60''E	3.28	81.80	Sub-humid	Temperate
Bouira_2	Bouira, Djbel Inesman	928	36°26'8.49''N	3°56'8.15''E	1.85	102.89	Humid	Temperate
Bouira_3	Bouira, Djbel Tegjda	1050	36°25'6.63''N	4°05'1.24''E	1.36	110.45	Humid	Temperate
Bouira_4	Bouira, Haizer	615	36°24'3.68''N	4°03'8.55''E	3.10	84.36	Sub-humid	Temperate
Bouira_5	Bouira, Oued El Zian	525	36°14'5.87''N	3°57'2.31''E	3.46	79.26	Sub-humid	Temperate
Boumerdes_1	Boumerdes, Corso	34	36°43'07.74''N	3°26'4.94''E	6.83	100.52	Humid	Temperate
Boumerdes_2	Boumerdes, Larbattache	81	36°39'09.73''N	3°20'7.16''E	6.64	103.67	Humid	Temperate
Boumerdes_3	Boumerdes, Thnia	50	36°44'06.67''N	3°30'5.70''E	6.76	101.59	Humid	Temperate
BouSaâda_1	BouSaâda, Djbel Azdin	591	35°14'8.62''N	4°09'9.95''E	3.20	25.97	Arid	Temperate
BouSaâda_2	BouSaâda, Djbel Messaad	1005	35°02'9.93''N	4°06'5.58''E	1.54	48.40	Semi-arid	Temperate
BouSaâda_3	BouSaâda, Djbel Messaad	999	35°02'9.75''N	4°06'5.84''E	1.57	48.06	Semi-arid	Temperate
Chlef_1	Chlef, Sendjas	536	35°59'0.20''N	1°30'2.04''E	4.82	97.66	Humid	Temperate
Chlef_2	Chlef, Sendjas	582	35°59'08.58''N	1°27'6.98''E	4.64	103.13	Humid	Temperate
Constantine_1	Constantine, Ain Zbera	720	36°13'7.21''N	6°31'4.03''E	2.26	44.45	Semi-arid	Temperate
Constantine_2	Constantine, Ainsmara	626	36°16'22.85''N	6°31'18.10'E	2.64	40.84	Semi-arid	Temperate
Constantine_3	Constantine, BéniHamidene	435	36°30'20.38''N	6°33'7.98''E	3.40	33.68	Semi-arid	Temperate

Constantine_4	Constantine, BéniHamidene	435	36°30'20.38''N	6°33'7.98''E	3.40	33.68	Semi-arid	Temperate
Constantine_5	Constantine, BéniHamidene	435	36°30'20.38''N	6°33'7.98''E	3.40	33.68	Semi-arid	Temperate
Constantine_6	Constantine, BéniHamidene	435	36°30'20.38''N	6°33'7.98''E	3.40	33.68	Semi-arid	Temperate
Constantine_7	Constantine, BéniHamidene	435	36°30'20.38''N	6°33'7.98''E	3.40	33.68	Semi-arid	Temperate
Constantine_8	Constantine, BéniHamidene	435	36°30'20.38''N	6°33'7.98''E	3.40	33.68	Semi-arid	Temperate
Constantine_9	Constantine, Djbel El Ouehch	969	36°24'4.13''N	6°41'1.84''E	1.26	54.25	Sub-humid	Temperate
Constantine_10	Constantine, Djbel El Ouehch	795	36°23'3.69''N	6°40'5.76''E	1.96	47.36	Semi-arid	Temperate
Constantine_11	Constantine, Djbel El Ouehch	787	36°23'2.85''N	6°40'6.19''E	1.99	47.05	Semi-arid	Temperate
Constantine_12	Constantine, El Mrij	731	36°20'3.98''N	6°41'2.03''E	2.22	44.87	Semi-arid	Temperate
Constantine_13	Constantine, El Mrij	745	36°20'2.41''N	6°41'2.15''E	2.16	45.41	Semi-arid	Temperate
Constantine_14	Constantine, Oued Ain Medjredane	563	36°18'17.48''N	6°34'24.46'E	2.89	38.46	Semi-arid	Temperate
Constantine_15	Constantine, Oued Ain Medjredane	563	36°18'17.48''N	6°34'24.46'E	2.89	38.46	Semi-arid	Temperate
Constantine_16	Constantine, Oued Ain Medjredane	563	36°18'17.48''N	6°34'24.46'E	2.89	38.46	Semi-arid	Temperate
El-Taref_1	El-Taref, Dréan	38	36°39'4.41''N	7°43'1.48''E	7.05	93.79	Humid	Warm winter
El-Taref_2	EL-Taref, Oued Sibousse	40	36°39'4.22''N	7°42'9.66''E	7.04	94.02	Humid	Warm winter
Guelma_1	Guelma, Autoroute	500	36°30'15.59''N	7°30'30.10E	3.51	78.65	Sub-humid	Temperate
Guelma_2	Guelma, Belkheir	173	36°28'30.99''N	7°28'58.42E	4.82	62.41	Sub-humid	Temperate
Guelma_3	Guelma, Belkheir	350	36°32'41.20''N	7°28'33.43E	4.11	71.08	Sub-humid	Temperate
Guelma_4	Guelma, Beni Mezline	261	36°28'37.85''N	7°36'18.10E	4.46	66.68	Sub-humid	Temperate
Guelma_5	Guelma, Guelaat Bou Sbaa	230	36°32'32.16''N	7°30'13.23E	4.59	65.17	Sub-humid	Temperate
Guelma_6	Guelma, Héliopolis	174	36°30'20.68''N	7°27'2.55''E	4.81	62.45	Sub-humid	Temperate
Jijel_1	Jijel, Milia	52	36°42'09.64''N	6°16'1.82''E	6.52	152.26	Humid	Temperate
Jijel_2	Jijel, Sibari	250	36°33'05.43''N	6°16'8.47''E	5.73	178.70	Humid	Temperate
Jijel_3	Jijel, Sidi Maarouf	93	36°37'01.25''N	6°16'3.35''E	6.36	157.63	Humid	Temperate
Jijel_4	Jijel, Taxanaa Zgroura	573	36°39'53.68''N	5°45'.3.77''E	4.44	224.75	Humid	Temperate
Jijel_5	Jijel, Taxanaa Zgroura	573	36°39'53.68''N	5°45'.3.77''E	4.44	224.75	Humid	Temperate
Jijel_6	Jijel, Taxanaa Zgroura	573	36°39'53.68''N	5°45'.3.77''E	4.44	224.75	Humid	Temperate
Jijel_7	Jijel, Taxanaa Zgroura	573	36°39'53.68''N	5°45'.3.77''E	4.44	224.75	Humid	Temperate
Khenchela_1	Khenchela, AinSilan	1133	35°26'1.28''N	7°05'2.39''E	1.40	61.56	Sub-humid	Temperate
Khenchela_2	Khenchela, AinSilan	1130	35°26'1.37''N	7°05'2.34''E	1.41	61.42	Sub-humid	Temperate
Khenchela_3	Khenchela, Djbel El Aamra	770	34°57'03.84''N	7°02'2.64''E	2.85	44.71	Semi-arid	Temperate

Khenchela_4	Khenchela, Djbel Laknif	1012	35°29'01.57"N	7°15'2.15"E	1.88	55.82	Sub-humid	Temperate
Khenchela_5	Khenchela, Djbel Laknif	1013	35°29'01.57"N	7°15'2.15"E	1.88	55.87	Sub-humid	Temperate
Khenchela_6	Khenchela, Djbel Laknif	1020	35°29'01.57"N	7°15'2.15"E	1.85	56.19	Sub-humid	Temperate
Khenchela_7	Khenchela, Djbelkais	1049	35°25'08.30"N	7°04'8.97"E	1.74	57.56	Sub-humid	Temperate
Khenchela_8	Khenchela, Hmamm El Salhin	1064	35°26'8.30"N	7°04'9.02"E	1.68	58.27	Sub-humid	Temperate
Khenchela_9	Khenchela, Zaouia	788	34°57'07.34"N	7°02'2.09"E	2.78	45.52	Semi-arid	Temperate
Laghouat_1	Laghouat, Djbel Gnoura El Sbihi	975	34°11'09.82"N	3°03'4.27"E	1.87	26.96	Arid	Temperate
Laghouat_2	Laghouat, Djbel Umm Al-Duloua	900	33°45'00.17"N	2°39'5.22"E	2.17	23.68	Arid	Temperate
Laghouat_3	Laghouat, Djbel Umm Al-Duloua	877	33°45'00.11"N	2°39'5.37"E	2.26	22.68	Arid	Temperate
Mascara_1	Mascara, Ben Ouali	178	35°32'05.84"N	0°21'8.39"W	5.53	28.63	Arid	Temperate
Mascara_2	Mascara, Oggaz	196	35°32'02.73"N	0°21'9.51"W	5.46	29.45	Arid	Temperate
Mascara_3	Mascara, Sig	74	35°33'01.62"N	0°15'0.32"W	5.95	23.95	Arid	Temperate
Médea_1	Médea, Berouaghia	826	36°08'1.67"N	2°52'3.10"E	4.32	80.90	Sub-humid	Temperate
Médea_2	Médea, Oued El Hmmam	818	36°09'4.40"N	2°57'6.18"E	4.35	80.46	Sub-humid	Temperate
Médea_3	Médea, Oued El Malah	615	36°12'0.00"N	3°08'6.59"E	5.16	69.63	Sub-humid	Temperate
Médea_4	Médea, Oued El Zeboudj	777	36°10'6.57"N	2°59'9.58"E	4.51	78.24	Sub-humid	Temperate
Mila_1	Mila, Amira Arrès	264	36°30'0.95"N	6°05'9.28"E	6.02	25.61	Arid	Temperate
Mila_2	Mila, Bel El Rjass	260	36°27'8.94"N	6°06'2.59"E	6.04	25.43	Arid	Temperate
Mila_3	Mila, Djbel Boucherf	755	36°27'4.26"N	6°00'2.82"E	4.06	49.21	Semi-arid	Temperate
Mila_4	Mila, Djbel Boucherf	776	36°27'1.76"N	6°00'5.06"E	3.98	50.27	Sub-humid	Temperate
Mila_5	Mila, Djbel El Kaf	603	36°32'2.49"N	5°59'7.66"E	4.67	41.66	Semi-arid	Temperate
Mila_6	Mila, Hmamm Bouarbia	603	36°32'0.29"N	6°00'8.43"E	4.67	41.66	Semi-arid	Temperate
Mila_7	Mila, Romani Stara	639	36°27'5.06"N	6°01'9.38"E	4.52	43.43	Semi-arid	Temperate
Mila_8	Mila, Sidi Khelifa	643	36°22'5.88"N	6°19'4.34"E	4.51	43.63	Semi-arid	Temperate
Mila_9	Mila, Zeraia Djbel Ben Htan	356	36°28'0.77"N	6°09'6.56"E	5.66	29.86	Arid	Temperate
M'sila_1	M'sila, Djbel Olad Hmed	1080	35°59'3.99"N	4°11'3.15"E	2.02	59.92	Sub-humid	Temperate
M'sila_2	M'sila, Driatte	958	35°58'1.24"N	4°26'1.25"E	2.50	52.47	Sub-humid	Temperate
M'sila_3	M'sila, Hammam Dalaa	943	35°58'6.18"N	4°23'8.18"E	2.56	51.57	Sub-humid	Temperate
M'sila_4	M'sila, Oued Lkman	599	35°49'5.03"N	5°10'4.80"E	3.94	31.74	Semi-arid	Temperate
Oran_1	Oran, Forêt De M'sila	436	35°40'02.48"N	0°47'152"W	7.28	128.37	Humid	Warm winter
Oran_2	Oran, Misserghin	217	35°38'06.32"N	0°45'2.89"W	8.16	90.91	Humid	Warm winter

Oran_3	Oran, Senia	212	35°38'06.98''N	0°42'0.27''W	8.18	90.08	Humid	Warm winter
OuedHammam	Oued Hammam El Biban	577	36°12'04.31''N	4°22'4.46''E	3.20	22.97	Arid	Temperate
OumElBouaghi_1	OumElBouaghi, Ain Zitoune	875	35°42'9.79''N	7°00'6.44''E	1.26	39.76	Semi-arid	Temperate
OumElBouaghi_2	OumElBouaghi, Djbel Lgriratte	859	35°42'9.71''N	7°00'6.90''E	1.32	39.06	Semi-arid	Temperate
OumElBouaghi_3	OumElBouaghi, Djbel Sidi Rgiss	1214	35°54'1.61''N	7°07'5.34''E	-0.10	55.16	Sub-humid	Cool winter
OumElBouaghi_4	OumElBouaghi, Foret	1003	35°52'9.90''N	7°07'3.97''E	0.74	45.47	Semi-arid	Temperate
Relizane_1	Relizane, Ain El Rahma	330	35°37'04.62''N	0°23'7.65''E	4.36	46.99	Semi-arid	Temperate
Relizane_2	Relizane, El Kalaa	471	35°35'07.77''N	0°20'2.65''E	3.80	53.62	Sub-humid	Temperate
Relizane_3	Relizane, El Kalaa	471	35°35'07.77''N	0°20'2.65''E	3.80	53.62	Sub-humid	Temperate
Relizane_4	Relizane, Zemmora	382	35°42'08.50''N	0°46'2.47''E	4.15	49.41	Semi-arid	Temperate
Sétif_1	Sétif, Beni Ourtilane	1109	36°25'07.73''N	4°53'1.84''E	-0.31	43.35	Semi-arid	Cool winter
Sétif_2	Sétif, Djbel Boutalab	854	35°41'4.09''N	5°10'4.80''E	0.71	32.49	Semi-arid	Temperate
Sétif_3	Sétif, El Nakhla	641	36°23'02.18''N	4°57'9.75''E	1.56	23.77	Arid	Temperate
Sétif_4	Sétif,Djbel Lazali	776	36°23'08.86''N	4°57'8.85''E	1.02	29.26	Arid	Temperate
SidiBel-Abbes_1	SidiBel-Abbes, Ain El Berd	516	35°22'07.93''N	0°29'8.04''W	2.64	40.60	Semi-arid	Temperate
SidiBel-Abbes_2	SidiBel-Abbes, Sidi Brahim	435	35°14'08.23''N	0°35'1.11''W	2.96	36.97	Semi-arid	Temperate
SidiBel-Abbes_3	SidiBel-Abbes, Sidi Hamadouch	419	35°17'08.34''N	0°33'1.97''W	3.02	36.26	Semi-arid	Temperate
Skikda_1	Skikda, Bni Tafout	39	36°42'09.43''N	7°18'8.63''E	8.75	140.38	Humid	Warm winter
Skikda_2	Skikda, Dachra Sad Zit El Anaba	46	36°41'06.65''N	7°19'3.94''E	8.72	141.48	Humid	Warm winter
Skikda_3	Skikda, Dachra Sad Zit El Anaba	46	36°41'06.65''N	7°19'3.94''E	8.72	141.48	Humid	Warm winter
Skikda_4	Skikda, Dachra Sad Zit El Anaba	46	36°41'06.65''N	7°19'3.94''E	8.72	141.48	Humid	Warm winter
Skikda_5	Skikda, Dachra Sad Zit El Anaba	46	36°41'06.65''N	7°19'3.94''E	8.72	141.48	Humid	Warm winter
Skikda_6	Skikda, Sidi Saîd	43	36°42'09.37''N	7°17'7.07''E	8.74	141.01	Humid	Warm winter
Skikda_7	Skikda, Sidi Saîd	43	36°42'09.37''N	7°17'7.07''E	8.74	141.01	Humid	Warm winter
Skikda_8	Skikda, Sidi Saîd	43	36°42'09.37''N	7°17'7.07''E	8.74	141.01	Humid	Warm winter
Skikda_9	Skikda, Sidi Saîd	43	36°42'09.37''N	7°17'7.07''E	8.74	141.01	Humid	Warm winter
Skikda_10	Skikda, Sidi Saîd	43	36°42'09.37''N	7°17'7.07''E	8.74	141.01	Humid	Warm winter
Skikda_11	Skikda, Sidi Saîd	43	36°42'09.37''N	7°17'7.07''E	8.74	141.01	Humid	Warm winter
Skikda_12	Skikda, Sidi Saîd	43	36°42'09.37''N	7°17'7.07''E	8.74	141.01	Humid	Warm winter
Skikda_13	Skikda, Sidi Saîd	43	36°42'09.37''N	7°17'7.07''E	8.74	141.01	Humid	Warm winter
Skikda_14	Skikda, Sidi Saîd	43	36°42'09.37''N	7°17'7.07''E	8.74	141.01	Humid	Warm winter

Skikda_15	Skikda, Sidi Saîd	43	36°42'09.37"N	7°17'7.07"E	8.74	141.01	Humid	Warm winter
Skikda_16	Skikda, Tabat Nwara	45	36°42'03.11"N	7°19'1.36"E	8.73	141.33	Humid	Warm winter
Skikda_17	Skikda, Tabat Nwara	45	36°42'03.11"N	7°19'1.36"E	8.73	141.33	Humid	Warm winter
Skikda_18	Skikda, Zana Sad Zit El Anba	118	36°40'06.42"N	7°17'4.97"E	8.44	152.98	Humid	Warm winter
Skikda_19	Skikda, Zana Sad Zit El Anba	118	36°40'06.42"N	7°17'4.97"E	8.44	152.98	Humid	Warm winter
Skikda_20	Skikda, Zana Sad Zit El Anba	118	36°40'06.42"N	7°17'4.97"E	8.44	152.98	Humid	Warm winter
Skikda_21	Skikda, Zana Sad Zit El Anba	118	36°40'06.42"N	7°17'4.97"E	8.73	141.33	Humid	Warm winter
Tébessa_1	Tébessa, Centre-Ville	546	36°14'7.99"N	7°57'2.37"E	1.95	74.46	Sub-humid	Temperate
Tébessa_2	Tébessa, Djbel Tazerbonette	1004	35°00'6.60"N	7°39'6.99"E	1.08	43.25	Semi-arid	Temperate
Tébessa_3	Tébessa, Laawinatte	691	35°49'6.20"N	7°52'3.07"E	2.33	29.37	Arid	Temperate
Tébessa_4	Tébessa, Laawinatte	694	35°50'4.15"N	7°52'1.92"E	2.32	29.50	Arid	Temperate
Tébessa_5	Tébessa, Zaarouria	895	36°11'8.49"N	7°57'1.60"E	0.56	92.86	Humid	Temperate
Tébessa_6	Tébessa, Zaarouria	887	36°11'8.54"N	7°57'2.31"E	0.59	92.42	Humid	Temperate
Telmcen_1	Telmcen, Hennaya	375	34°58'00.96"N	1°22'00.5"W	5.08	56.89	Sub-humid	Temperate
Telmcen_2	Telmcen, Remchi	119	35°08'00.68"N	1°26'8.65"W	6.11	30.88	Semi-arid	Temperate
Tipaza_1	Tipaza, Pent Mzfrane	18	36°41'02.34"N	2°47'9.37"E	8.42	96.33	Humid	Warm winter
Tipaza_2	Tipaza, Plage Chenoua	45	36°37'02.42"N	2°24'4.38"E	8.31	98.33	Humid	Warm winter
Tipaza_3	Tipaza, Sidi Rached	146	36°34'08.00"N	2°29'4.85"E	7.90	105.98	Humid	Warm winter
Tissemsilt_1	Tissemsilt, Tnite El Had	931	35°54'7.61"N	2°04'0.32"E	1.97	53.67	Sub-humid	Temperate
Tissemsilt_2	Tissemsilt, Youssoufia	817	35°57'1.20"N	2°06'3.73"E	2.43	47.74	Semi-arid	Temperate
TiziOuzou_1	Tizi Ouzou, Beni Yenni	565	36°33'07.42"N	4°12'9.04"E	5.09	124.31	Humid	Temperate
TiziOuzou_2	Tizi Ouzou, Djbel Djurdjura	1001	36°29'09.96"N	4°14'7.96"E	3.35	152.53	Humid	Temperate
TiziOuzou_3	Tizi Ouzou, Maatkas	87	36°42'02.15"N	4°00'3.36"E	7.00	96.23	Humid	Warm winter
TiziOuzou_4	Tizi Ouzou, Ouacif	232	36°35'00.32"N	4°09'5.67"E	6.42	104.45	Humid	Temperate

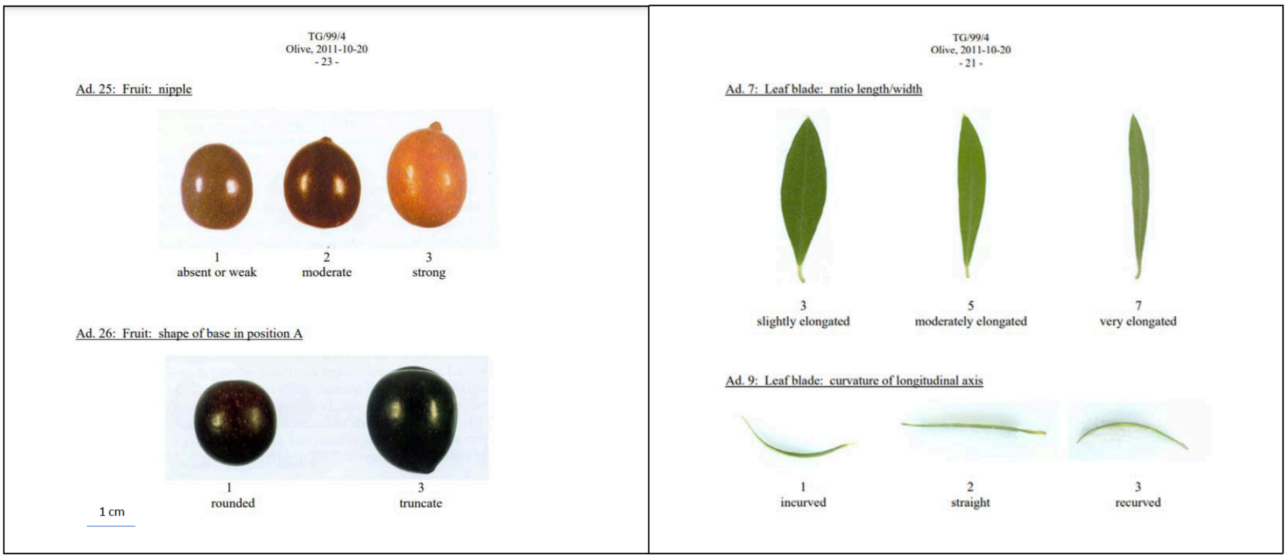

Supplementary Figure S1. An example of some descriptors indicated by UPOV for morphological characterization of olive.

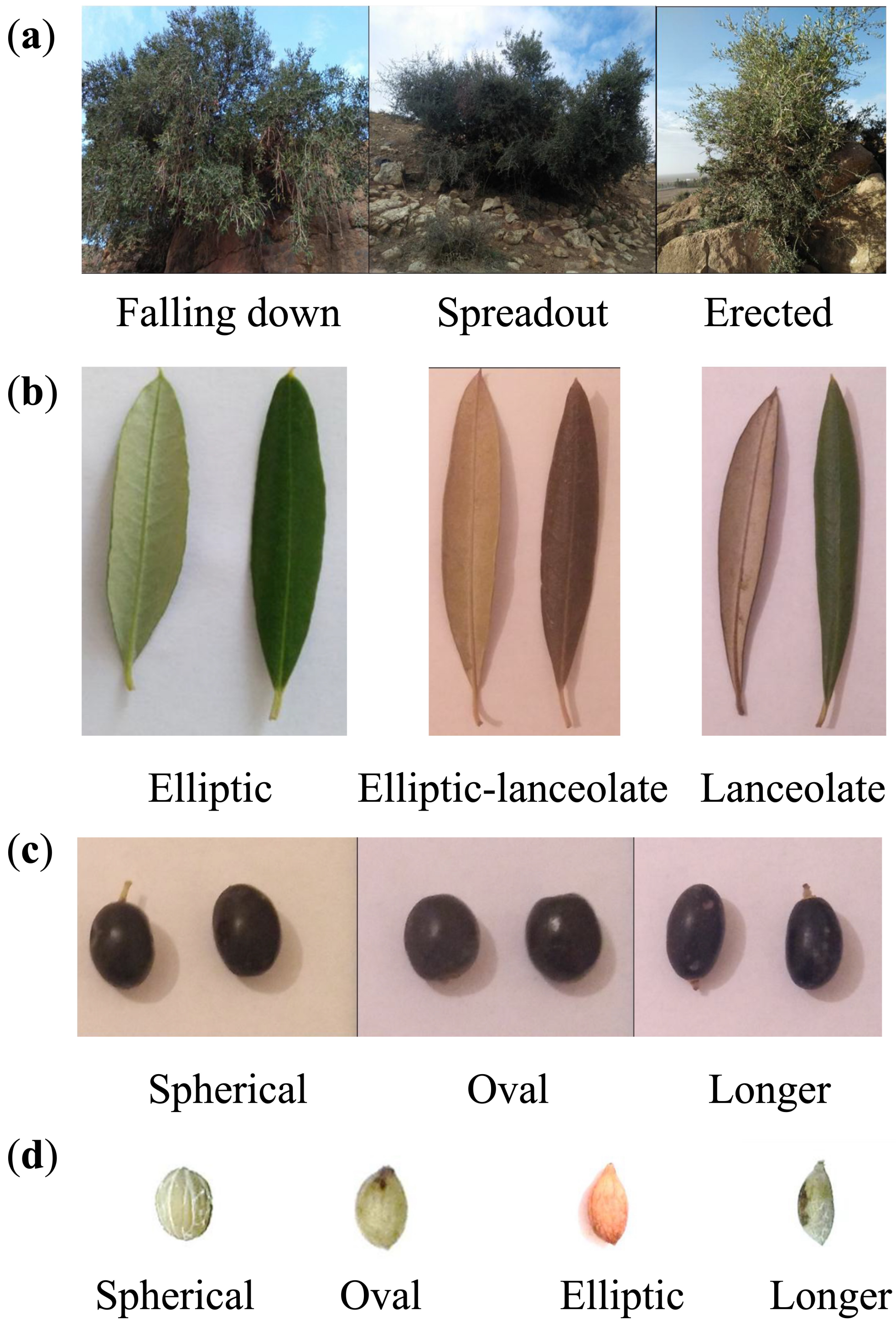

Supplementary Figure S2. Pictures representing some of the qualitative traits of the samples analyzed in this study. The tree habit (**a**) leaf shape (**b**), fruit shape (**c**) and stone shape (**d**) are shown.
